# Impact of a Static Magnetic Field on Early Osseointegration: A Pilot Study in Canines

**DOI:** 10.3390/ma16051846

**Published:** 2023-02-23

**Authors:** Roberta Michels, Carina Kampleitner, Toni Dobsak, Kevin Doppelmayer, Patrick Heimel, Stefan Lettner, Stefan Tangl, Reinhard Gruber, César Augusto Magalhães Benfatti

**Affiliations:** 1 Center for Research on Dental Implants (CEPID), School of Dentistry (ODT), Universidade Federal de Santa Catarina (UFSC), Florianópolis 88036-020, Brazil; 2 Robert K. Schenk Laboratory of Oral Histology, Department of Periodontology, School of Dental Medicine, University of Bern, 3010 Bern, Switzerland; 3 Karl Donath Laboratory for Hard Tissue and Biomaterial Research, University Clinic of Dentistry, Medical University of Vienna, 1090 Vienna, Austria; 4 Ludwig Boltzmann Institute for Traumatology, The Research Center in Cooperation with AUVA, 1200 Vienna, Austria; 5 Austrian Cluster for Tissue Regeneration, 1200 Vienna, Austria; 6 Department of Oral Biology, Medical University of Vienna, 1090 Vienna, Austria

**Keywords:** static magnetic field, osseointegration, dental implant, animal study

## Abstract

A static magnetic field generated by neodymium–iron–boron (NdFeB) magnets placed in the inner cavity of dental implants can enhance bone regeneration in rabbits. It is, however, unknown whether static magnetic fields support osseointegration in a canine model. We therefore determined the potential osteogenic effect of implants carrying NdFeB magnets inserted in the tibia of six adult canines in the early stages of osseointegration. Here, we report that after 15 days of healing, magnetic and regular implants showed a high variation with a median new bone-to-implant contact (nBIC) in the cortical (41.3% and 7.3%) and the medullary (28.6% and 44.8%) region, respectively. Consistently, the median new bone volume/tissue volume (nBV/TV) in the cortical (14.9% and 5.4%) and the medullary (22.2% and 22.4%) region were not significantly different. One week of healing only resulted in negligible bone formation. These findings suggest that considering the large variation and the pilot nature of this study, magnetic implants failed to support peri-implant bone formation in a canine model.

## 1. Introduction

Dental implants have become a standard of care to replace missing teeth [1]. It is the osseointegration, the consolidation of the titanium screws within the surrounding bone, that provides a vital anchoring of the dental implants in the jaw [2]. Osseointegration thus depends on the process of bone regeneration, in particular intramembranous ossification, where blood vessels bring in the osteogenic cells that later differentiate into bone-forming osteoblasts [3,4]. Bone is also a source of osteoblasts and their progenitors, which may explain the osteogenic properties of autografts [5]. The osteoblasts initially form an immature woven bone that is later enforced into plexiform bone which ultimately undergoes remodeling into the mature lamellar bone [6]. Since osseointegration of a dental implant depends on bone regeneration, research attempts go towards the stimulation of local bone regeneration, the bone immediately adjacent to the threads of the implants.

Magnetic fields have traditionally been implicated to support the natural process of bone regeneration, particularly with respect to fracture healing [7,8]. Recently, accumulating evidence has provided insights into how static magnetic fields from permanent magnets and pulsed electromagnetic fields affect the process of osseointegration of dental implants [9,10]. For instance, magnetic flux may stimulate osseointegration by increasing blood circulation and consequently enhancing the supply of oxygen and nutrients or by modulating cellular activity and signaling pathways (e.g., Wnt, MAPK signaling) relevant to bone formation [9,10]. In a clinical study, magnetic rather than conventional healing caps increased the implant stability quotient over time [11]. Implant stability quotients further identified a significantly higher implant stability of a static magnetic field compared to conventional implants 1 month after placement [12], a finding that was confirmed by 1-, 2-, and 3-month observation periods [13]. Taken together, there is reason to suggest that local magnetic fields overall cause a gain in secondary stability of the dental implants in a clinical scenario. However, it requires preclinical research to gain insights into the cellular aspects of how magnetic fields support osseointegration.

Preclinical research overall supports the beneficial effects of magnetic fields with respect to osseointegration, as has been elegantly summarized in two recent reviews [9,10]. Static magnetic fields were tested for their impact on osseointegration in rats, rabbits, and dogs. Histological and radiological methods quantified the amount, the distribution, and the maturity of the peri-implant newly formed bone. For instance, magnets moderately supported osseointegration in rabbits after 1, 2, 4, and 8 weeks of healing [14,15], and observation periods of 12 weeks were also reported [16]. Overall, the results indicate a moderate increase in osseointegration linked to the static magnetic field. Consistently, in a dog model, with static magnets increased osseointegration parameters at 8- and 12-week observation periods [17]. However, the impact of a static magnetic field on early osseointegration in a dog tibia implantation model remains unknown.

This pilot study aimed to determine the impact of a static magnetic field on the early stages of osseointegration following up on the insights provided with the dog model. We report here that implants carrying neodymium–iron–boron (NdFeB) magnets could not initiate a considerable amount of new bone formation after 7 days. After 15 days of healing, there was a trend that magnets support median cortical osseointegration but with a high variance when compared to regular non-magnetic implants.

## 2. Materials and Methods

### 2.1. Fabrication of Neodymium–Iron–Boron (NdFeB) Magnetic Implants

**Figure 1:**
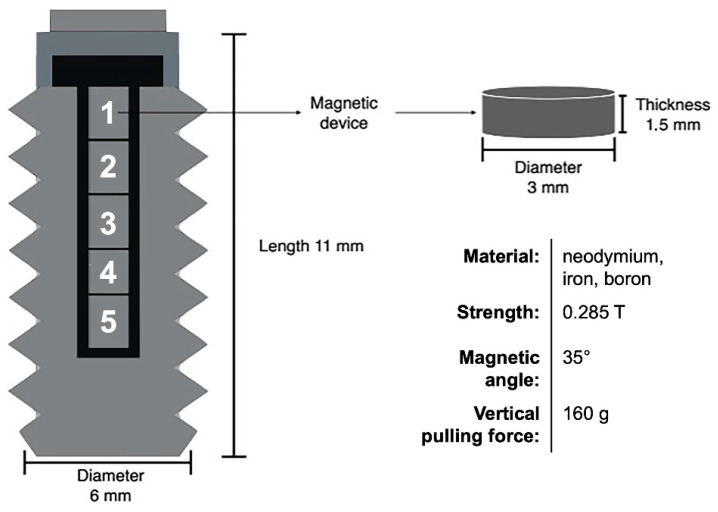
Schematic illustration and features of the titanium implants carrying five installed magnetic bars made of neodymium (Nd), iron (Fe), and boron (B). Tesla (T) is the unit of magnetic flux density. Magnetic welding angles were fixed at 35°.

Custom-made sterile dental implants made of commercially pure titanium bars were machined with a diameter of 6.0 mm and a length of 11.5 mm, as recently reported [18]. Implants were subjected to repeated cleaning cycles with detergent, distilled water, and drying at 60 °C. The passivation was performed according to the ASTM (American Society for Testing and Materials) F86 standards for removing free iron and contaminants. For the magnetic implants, five bars of neodymium (Nd), iron (Fe), and boron (B) were installed in the core of the construct, as illustrated in Figure 1.

### 2.2. Study Design

Animal experiments were approved by the Ethics Committee on the Use of Animals (CEUA) of the Federal University of Santa Catarina (114/CEUA/PRPe/2008) and performed in accordance with the ARRIVE guidelines. Six 12-month-old male beagle dogs with an average weight of 15 kg were randomly assigned into two treatment groups evaluated at 7- or 15-days post-surgery. Each animal received in total five implants divided into both tibial bones, resulting in two to three implants of the same treatment group, either magnetic or regular implants, per implantation site.

### 2.3. Surgical Procedure

The surgical procedure has been previously described by Bins-Ely et al. [18]. Briefly, the animals received an intramuscular (i.m.) injection of 0.44 mg/kg atropine sulfate (Atropinon^®^, Hipolabor Farmacêutica Ltd., Borges/Sabará, Brazil) followed by administration of 16 mg/kg ketamine hydrochloride (Francotar^®^, Virbac, Saúde Animal, Vila Hamburguesa, Brazil) and 3 mg/kg xylazine (Rompun^®^, Bayer SA, Leverkusen, Germany) (both i.m.) for general anesthesia. First, a longitudinal incision was made above the tibia to expose the underlying bone. Then a perforation was created using a surgical drill under constant irrigation with saline solution. Two to three sterile implants (length = 11.5 mm, Ø = 6.0 mm) of the same treatment group, either magnetic or regular implants, were subsequently implanted per site below the tibial crest into the diaphysis of the tibia. Finally, the incision was sutured in two layers using an absorbable 5.0 vicryl (intern) (Ethicon-Vicryl^®^, Johnson & Johnson, São Paulo, Brazil) and a 4.0 nylon thread (extern) (Somerville^®^, Jaboatao dos Guararapes, Brazil). The animals were housed individually during the study period and euthanized 7 or 15 days after implant placement with a lethal dose of sodium thiopental (Thioembutal^®^, Sao Bernardo do Campo, Brazil).

### 2.4. Histological Analysis

After 7 or 15 days, tibiae were harvested and dissected to analyze each implant individually. Formalin-fixed (10%) samples were dehydrated in ascending grades of ethanol and embedded in LR white resin (Sigma-Aldrich, Saint Louis, MO, USA). Blocks were further processed using the EXAKT cutting and grinding equipment (EXAKT Apparatebau, Norderstedt, Germany). Undecalcified thin-ground sections were prepared through the center of the implant and stained with Levai–Laczko dye, a variant of the Giemsa dye that allows distinguishing reliably between old bone, newly formed bone, and bone debris resulting from the surgical procedure and implant placement. In this staining, woven bone appears dark pink, mature bone light pink, and soft tissue blue. The slices were scanned using an Olympus BX61VS digital virtual microscopy system (DotSlide 2.4, Olympus, Tokyo, Japan) with 20× magnification at a resolution of 0.32 μm per pixel.

### 2.5. Histomorphometric Analysis

**Figure 2:**
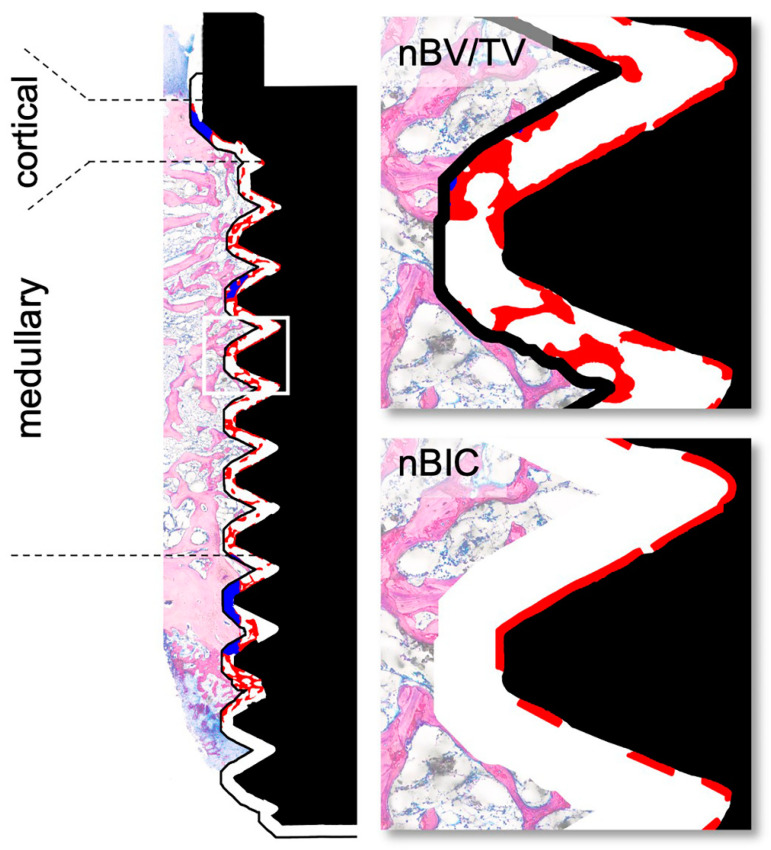
Representation of the histomorphometrically analyzed regions of interest. The peri-implant tissue was divided into two compartments—the cortical and medullary regions. In both, we distinguished between the new (red) and old (blue) bone within 200 µm adjacent to the implant surface to evaluate new bone formation (= new bone volume per tissue volume; nBV/TV). We also assessed the new bone-to-implant contact (nBIC) to present the relative coverage of the implant surface by newly formed bone.

Histomorphometric analysis was blinded and performed at two regions of interest, representing the cortical and medullary compartments. The respective areas were manually segmented, and we classified newly formed bone and old bone using Adobe Photoshop (Adobe Inc., Mountain View, CA, USA). New bone volume per tissue volume (nBV/TV) within 200 µm adjacent to the implant surface and new bone-to-implant contact (nBIC) were determined and are presented as percentages (Figure 2). All measurements were taken using Definiens Developer XD 2.7 (Definiens AG, Munich, Germany).

### 2.6. Statistical Analysis

Histomorphometric results were graphed using GraphPad Prism 9.0 (GraphPad Software, Boston, MA, USA) and data are presented as median and range. One animal was excluded from the analysis due to a fractured paw, which was not associated with the surgical procedure. For induction, we used Welch’s two sample *t*-tests to compare treatments lasting 15 days per site for nBV/TV and nBIC. Descriptive statistics for nBV/TV and nBIC are shown in Supplementary Table S1. All calculations were made using R version 4.0.3 (R Core Team 2020). 

## 3. Results

### 3.1. Impact of Magnetic Field on the First Week of Osseointegration—Histology

**Figure 3:**
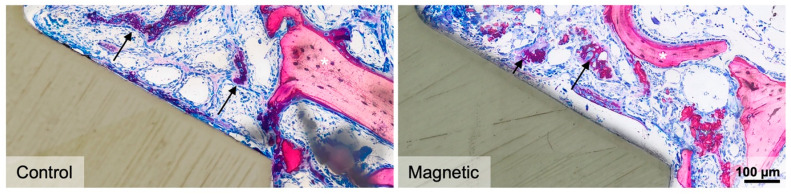
Histological analysis 7 days after implant placement. High-magnification images of representative thin-ground sections stained with Levai–Laczko dye present negligible woven bone formation (dark pink) in the medullary compartment of magnetic and regular implants (control). Black arrows denote newly formed bone, while white asterisks indicate pristine bone stained in light pink.

Consistent with our previous studies, osseointegration remains at the level of primary implant stability [6]. This principle may be overcome by the magnetic field. We first investigated the possible role of magnetic fields to enhance the initial stages of bone formation by analyzing the early 7-day observation period. Histological analysis showed consolidated granulation tissue indicated by the sprouting of nascent blood vessels that are embedded in an extracellular matrix. Besides fragments of the pristine bone resulting from the implant placement, only a negligible amount of new bone, indicated by the dark pink staining, was visible or even in contact with the implant surface (Figure 3). There was no obvious impact of the magnetic field on these peri-implant regions of interest.

### 3.2. Impact of Magnetic Field on the First Week of Osseointegration—Histomorphometry

**Figure 4:**
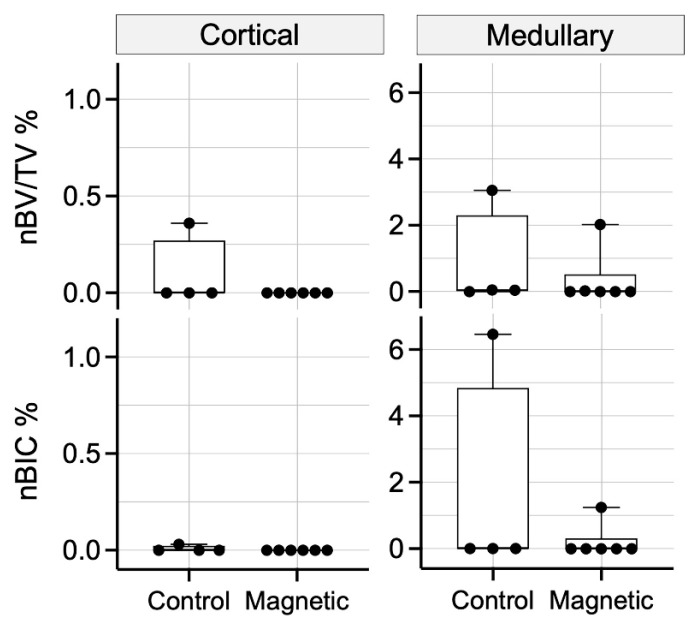
Histomorphometric analysis of osseointegration parameters after 7 days of healing in the cortical and medullary compartment. nBV/TV was determined within 200 µm adjacent to the implant surface. nBIC was measured to demonstrate the relative coverage of the implant by newly formed bone. Data are presented as median and range (*n* = 4–6). Control: regular implants.

To quantify the peri-implant new bone, histomorphometric analysis was performed. Consistent with the histological observations, histomorphometry revealed that magnetic and regular dental implants behaved similarly with only insignificant new bone formation in the cortical and medullary compartment resulting in a median nBV/TV and nBIC at zero for both treatment groups (Figure 4). Taken together, magnetic implants cannot overcome the delay between implant insertion and its primary stability to the secondary stability caused by new bone formation.

### 3.3. Impact of Magnetic Field on the Second Week of Osseointegration—Histology

**Figure 5:**
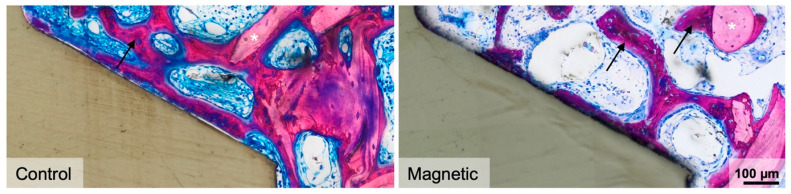
Histological analysis 15 days after implant placement. Representative images show high-magnification close-ups of the medullary compartment. Newly formed bone (dark pink) was evident in the peri-implant zone and at the implant surface forming a trabecular network that ultimately reconnected with pre-existing old bone (light pink). Black arrows denote woven bone, while white asterisks indicate pristine bone. Control: regular implants.

**Figure 6:**
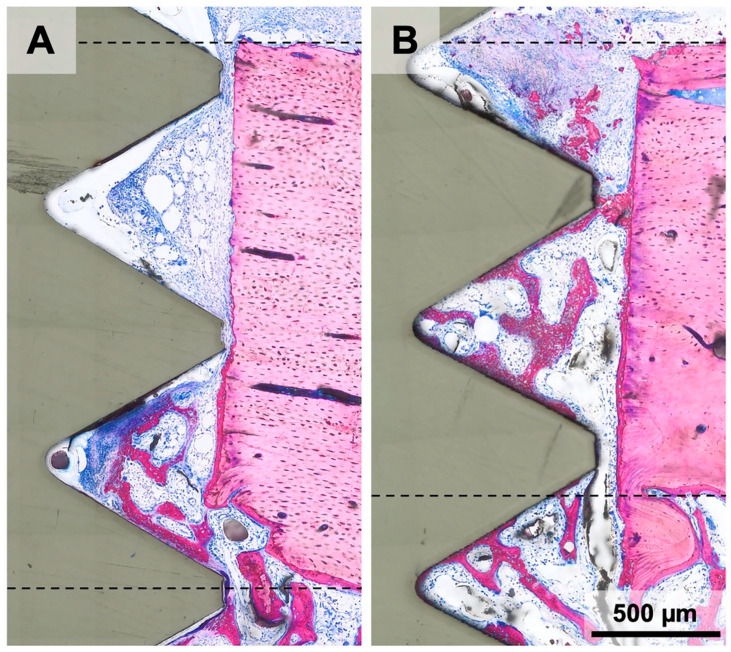
Histological images of the cortical compartment in magnetic field implants 15 days after implant insertion. (**A**) The thread of the implant hinders the sprouting of woven bone from the medullary cavity towards the periosteal side. (**B**) Implant threads are spaced from the pristine cortical bone, where a thin layer of new bone originating from the medullary region covers almost the entire thickness of the cortical bone and new bone spreads increasingly into the cortical peri-implant bone area. Dashed lines demonstrate the borders of the cortical compartment.

We then determined the impact of magnetic fields on bone formation 15 days after implant insertion. Histological analysis revealed a robust initiation of new bone formation with the expected features of a rapidly growing woven bone. This includes the formation of an interconnected trabecular network of mineralized structures with the osteoblast seams that are formed around the blood vessel (Figure 5). In the cortical bone area one might get the impression that once the threads are in a press-fit position with the bone, the bone marrow is sealed off. The consequence is that there is only little new bone formation in the cortical peri-implant bone area. However, when there is access to the medullary compartment, new bone sprouts into the peri-implant cortical space. This is particularly the case in magnetic field implants (Figure 6).

### 3.4. Impact of Magnetic Field on the Second Week of Osseointegration—Histomorphometry

**Figure 7:**
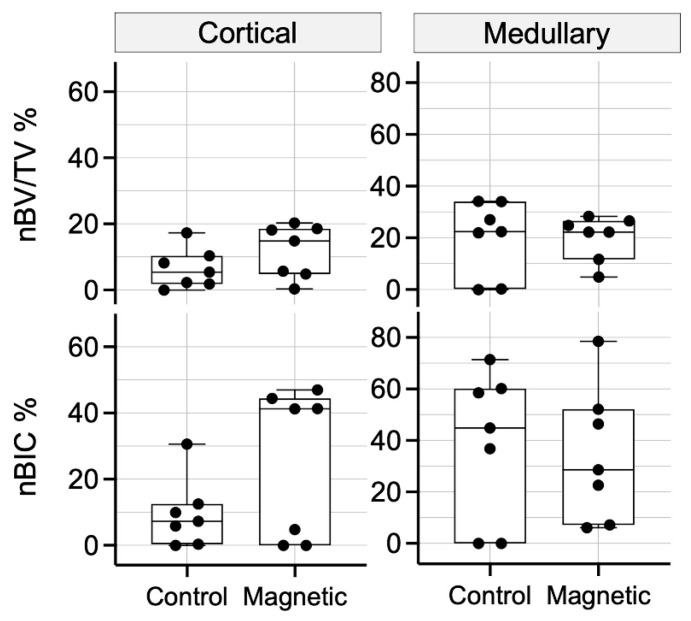
Histomorphometric analysis of osseointegration parameters after 15 days of healing in the cortical and medullary compartments. Graph demonstrates nBV/TV within 200 µm adjacent to the implant surface and the relative coverage of the implant with newly formed bone (nBIC). Data are presented as median and range (*n* = 7). Control: regular implants.

To calculate the impact of magnetic fields on peri-implant bone formation 15 days after implant insertion, histomorphometry was performed. Histomorphometry showed that the median cortical nBV/TV (14.9% and 5.4%; *p* = 0.188) and nBIC (41.3% and 7.3%; *p* = 0.123) were higher in the magnetic when compared to the regular dental implants, respectively (Figure 7). The distribution of the new bone formation reflects what was already observed with descriptive histological analysis. In the magnetic field group, four specimens had substantial new peri-implant bone formation, while another three specimens were almost lacking new bone formation. Thus, care should be taken when subjecting these specimens to a statistical analysis that overall suggests no effect of the magnetic field on cortical new bone formation. However, in all four specimens showing bone formation, the magnitude was higher than the average bone formation in the regular implants. In the medullary compartment, magnetic implants showed a comparable nBV/TV (22.2% and 22.4%; *p* = 0.984), but lower nBIC (28.6% and 44.8%; *p* = 0.774), in contrast to regular implants (Figure 7). Similar to the cortical area, there was a high variation in the medullary region because two specimens in each group showed only little new bone formation.

## 4. Discussion

This research was inspired by the increasing recognition of magnetic fields to support the process of osseointegration, which is mainly a consequence of the conserved sequence of early intramembranous bone formation, similar to what was proposed for classical primary fracture healing, in which bone ends are reconnected without the formation of a callus. Support for our research aim comes from two recent reviews that have summarized the relevant knowledge based on a large spectrum of interdisciplinary studies, suggesting a beneficial effect of static as well as pulsed electromagnetic fields on osseointegration [9,10]. Apart from all the rabbit studies [14,15,16], one dog model was applied to show that static magnets increased osseointegration parameters at 8- and 12-week observation periods [17]. In an extension of this research, we have focused on the very early impact of a static magnetic field on osseointegration in a dog tibia implantation model.

Classical treatment protocols in implant dentistry require 3 to 6 months of a load-free period after implant placement. Attempts have been made to shorten this healing phase with immediate implant placement [19] and loading protocols [20] in combination with innovative strategies to improve osseointegration. However, previous research has shown that there is no considerable amount of new bone at early time points after implantation that could be considered as secondary implant stability [6]. A static magnetic field might overcome that problem and reduce the time to achieve a vital anchorage. The clinical request for rapid healing and loading emphasizes our focus on the early stages of osseointegration over the long-term results where we expect a good integration for the tested machined titanium implants independent of the static magnetic field. Therefore, our interest was specifically directed towards the early healing phase between negligible to boosted bone formation and if a static magnetic field can initiate osseointegration at one and two weeks post-implantation.

The main finding of our study was that implants carrying NdFeB magnets failed to kick-start the natural process of bone formation at the early one-week observation period, even though signs of new bone formation were occasionally visible. Nevertheless, the magnetic field had no significant effect on either cortical or medullary peri-implant bone formation. However, after 15 days of healing, the situation became more complex as there was an obvious discrepancy in new bone formation in the cortical compartment. It seems that sealing off the medullary compartment with the press-fit mediated friction of the implant threads transiently blocks the ingrowth of new woven bone originating from the blood vessel sprouts of the medullary cavity. It is thus hard to blame any changes in bone formation on the magnetic fields. Nevertheless, we are left with the impression that once bone formation occurs, the presence of the magnetic field might be beneficial. However, this remains at the level of speculation and opens the door for future research with refined protocols.

The clinical relevance remains vague and should be restricted to the basic principles of bone regeneration, thus supporting the existing knowledge that there is a boost in bone formation and bone regeneration between one- and two-weeks following implant placement or defect healing in general. We are aware of the critical limitation that the tibia is an ectopic site for the placement of dental implants because implants were not inserted at sites of extracted teeth. Therefore, this study does not particularly simulate a clinical situation of immediate implant placement and should be interpreted with caution. However, long-bone tibial models in canines are commonly used in implant dentistry. The tibial diaphysis offers a large quantity of bone to study bone regeneration in response to human-sized dental implants and, similar to the alveolar bone, the implant is inserted into pure lamellar bone [21]. Outside of dental implant research, our findings might be of relevance to other fields, for instance in orthopedics, where screws and nails are inserted in the cortical bone and the medullary compartment. Thus, the data are of interest when applying implants with and without magnetic fields in orthopedic and trauma surgery.

There are obviously limitations that need to be acknowledged. Apart from the ectopic placement of the implants in the tibia and not the alveolar bone, the histological analysis revealed a considerable heterogeneity of the implant bed that stems from anatomical variation but also from the insertion of the implants. In the histological analysis, implants appeared to be placed mono- or bicortically. Some had an exact match with the drilling canal while others were placed leaving some scape open towards the medullary cavity. Overall, these variables accumulated and at least partially explain the high variance of parameters of osseointegration in both groups, independent of the presence or absence of the magnets. Therefore, conclusions regarding a possible influence of the magnetic field on the early stages of osseointegration can only be cautiously drawn. Thus, future research should aim to analyze osseointegration of magnetic implants placed in extraction sockets and maybe even consider a prosthetic loading protocol. Future research should also consider the option of a high-resolution micro-computed tomography analysis to gain more knowledge on the anatomical position, even though the interference of the titanium with the radiological beams has to be considered.

## 5. Conclusions

Taken together, the strength of this study is that it touches on the important early healing period covering the gap between almost no bone formation and a boost in bone formation with a focus on the impact of magnets. Our study indicates that implants carrying NdFeB magnets failed to initiate bone formation at the early one-week period. However, we observed a trend that magnetic implants support the cortical osseointegration after 15 days. Nevertheless, there is the weakness of the high variation that hinders us from drawing strong conclusions on the impact of static magnetic fields on early osseointegration in a cortical bone in dogs.

## Data Availability

The data presented in this study are available on request from the corresponding authors.

## Supplementary Materials

The following supporting information can be downloaded at: https://www.mdpi.com/article/10.3390/ma16051846/s1, Table S1: Descriptive statistics for the early osseointegration at 7- and 15-days post-implantation. Explants carrying the implant were histomorphometrically analyzed in two regions of interest, cortical and medullary compartments, for new bone formation (nBV/TV %) and relative coverage of the implant by newly formed bone (nBIC %).
