# Supplementary material for: Impact of a Static Magnetic Field on Early Osseointegration: A Pilot Study in Canines
Source: Materials (Basel). 2023 Feb 23;16(5):1846. doi: 10.3390/ma16051846 (PMC10003792; doi:10.3390/ma16051846)
Supplement: Supplementary file 1 [file materials-16-01846-s001.zip › materials-2163672-supplementary.pdf]

Supplementary Table S1: Descriptive statistics for the early osseointegration at 7- and 15-days post-implantation. Explants carrying the implant were histomorphometrically analyzed in two regions of interest, cortical and medullary compartment, for new bone formation (nBV/TV %) and relative coverage of the implant by newly formed bone (nBIC %).

			**nBV/TV %**			**nBIC %**			
Duration	Site	Treatment	Mean	SD	Median	Mean	SD	Median	n
7 days	Cortical	Control	0.1	0.2	0.0	0.0	0.0	0.0	4
		Magnetic	0.0	0.0	0.0	0.0	0.0	0.0	6
	Medullary	Control	0.8	1.5	0.0	1.6	3.2	0.0	4
		Magnetic	0.3	0.8	0.0	0.2	0.5	0.0	6
15 days	Cortical	Control	6.5	6.0	5.4	9.5	10.4	7.3	7
		Magnetic	11.8	8.0	14.9	25.5	22.5	41.3	7
	Medullary	Control	20.0	14.4	22.4	38.8	28.8	44.8	7
		Magnetic	20.1	8.6	22.2	34.5	26.2	28.6	7
